# A novel “three-dimensional-printed individual guide template-assisted percutaneous vertebroplasty” for osteoporotic vertebral compression fracture: a prospective, controlled study

**DOI:** 10.1186/s13018-021-02471-w

**Published:** 2021-05-20

**Authors:** Pei Lun Hu, Ji Sheng Lin, Hai Meng, Nan Su, Yong Yang, Qi Fei

**Affiliations:** Department of Orthopedics, Beijing Friendship Hospital, Capital Medical University, Xicheng District, Beijing, People’s Republic of China

**Keywords:** Osteoporotic vertebral compression fractures, Percutaneous vertebroplasty, Materialise Interactive Medical Image Control System, Three-dimensional printing technology, Navigation template

## Abstract

**Background:**

Conventional percutaneous vertebroplasty (PVP) are mainly guided by C-arm fluoroscopy, and it usually leads to excessive X-ray radiation exposure to patients, surgeons, and anesthetists. Moreover, multi-time fluoroscope may prolong the operation time. 3D-printed template could help minimize fluoroscopy shot times and fluoroscopy dosage during operation, and shorten operation time. We perform this study to compare the efficacy and accuracy of PVP assisted by “three-dimensional printed individual guide template” versus conventional PVP.

**Method:**

Patients who suffered acute painful single segment osteoporotic vertebral compression fracture(OVCF) needed operative treatment were randomly assigned into three-dimensional printing individual guide template-assisted percutaneous vertebroplasty group (group A) or conventional PVP guided by C-arm fluoroscopy group (group B) at a 1:1 ratio. Fluoroscopy times for puncture points (FTPP), total radiation dosages (TRD), total fluoroscopy time (TFT), and total operation time (TOT) were recorded as the main evaluation factors to evaluate the two operation procedures.

**Results:**

A total of 36 acute painful single segment OVCF patients were successfully operated on, and each group has 18 patients. None of the patients presented symptomatic complications. The surgical success rate in group A was 94.4%(17/18), one patient in the group A was failed and then operated by conventional procedure. FTPP (1.8 ± 0.8 in group A vs 5.2 ± 1.9 in group B, *P* < 0.05), TRD (4.9 ± 0.9 mGy vs 7.9 ± 1.6 mGy, *P* < 0.05), TFT (16.7 ± 2.9 vs 26.6 ± 5.3, *P* < 0.05), and total operation time (19.4 ± 2.4 min vs 27.8 ± 4.0 min, *P* < 0.05) were presented statistically difference in the two groups. The incidence of cement leakage occurred in group A (3/18, 16.7%) was less than that occurred in group B (7/18, 38.9%) (*P* > 0.05).

**Conclusions:**

Compared with the conventional PVP, “three-dimensional-printed individual guide template-assisted PVP” could minimize fluoroscopy shot times during operation and fluoroscopy dosage, shorten operation time, and is a more precise and feasible operation method.

**Trial registration:**

The present study was registered with the Chinese Clinical Trial Registry (ChiCTR) (http://www.chictr.org.cn), and its registration no. is ChiCTR1900024283.

## Introduction

With aging of the population, osteoporosis becomes increasingly common in the society. One of the most common problems with osteoporosis is the osteoporotic vertebral compression fractures (OVCFs). It is reported that OVCFs affect around 1.4 million patients in the world annually [1, 2]. As OVCFs could lead to persistent back pain and long-term morbidity [3, 4], making it a highly concerning clinical problem nowadays. Since 1987, Galibert et al. [5] performed the percutaneous vertebroplasty (PVP) for the treatment of an aggressive vertebral hemangioma; this minimally invasive surgery method that injects polymethylmethacrylate (PMMA) into the fractured vertebral body became one of the standard treatment measures for VCFs [6].

PVP is considered an effective treatment for the back pain caused by OVCFs [7–11]. However, the accuracy of the surgery mainly depends on the surgeons’ experience and multiple C-arm fluoroscopes during a conventional procedure. Thus, this procedure has the following drawbacks: (1) during conventional PVP, repeated fluoroscopes are needed to determine the optimal puncture points and to adjust the entry angles of puncture needles, which could lead to excessive X-ray radiation exposure to patients, surgeons, and anesthetists. According to a research of Niki T. Fitousi et al., throughout a traditional PVP procedure, the mean exposure dose of the surgeon’s hands was 1.661 mGy. This finding suggests that one surgeon could only perform around 150 PVP operations annually, without exceeding the annual safe dose constraint value [12]. Roger Harstall et al. reported that fluoroscopic imaging during percutaneous vertebroplasty requires longer beam-on-times than in other orthopedic routine procedures; this places surgeons under a higher risk of developing a thyroid cancer, statistically 25 times higher than the general population [13]. (2) Puncture-related complications of PVP were not rare. Almost 50% of patients have local complications from traditional PVP, with 95% of complications coming from cement leakage into surrounding tissues (paravertebral soft tissue, intervertebral disk, spinal canal, etc.), nerve root injury, and intra-spinal hematoma [14–18]. (3) For young surgeons, the learning curve to master the percutaneous pedicle puncture technique is quite long. (4) For complicated OVCF, like patient with severe osteoporosis, severe kyphosis, scoliosis, or multi-segment fractured vertebra, it commonly needs much more time and fluoroscope during operation procedure. Moreover, for elderly patients, they may not be capable of staying in prone operation position for such a long time.

Recently, various techniques have been introduced to ensure the safety and accuracy of the PVP [19, 20]. Previously, our team already made modifications of the conventional PVP procedures. In one published article [21], based on the preoperative CT images, we reconstructed the compressed vertebra, simulated the procedures in the software, and recorded all the data (punctuation depth, punctuation angle etc.), via measured those data to determine the punctuation plan during the real operation, time duration of the operation were shortened. After that, we made further improvements of the technique and introduced the 3D-printed template into the operation, which simplified surgical procedures by minimized the time of data measurements during operation. Our team had finished two pre-experimental cases of PVP assisted by 3D-printed guide templates and got success [7, 8]. Building upon the previous innovated operation research on the 3D-printed guide template assisted the PVP, we optimized the operation procedures. This study was then developed to explore if applying 3D printing guide template to PVP could indeed make the surgical procedure more precise, decrease the radiation exposure dosages, and shorten the operation time.

## Materials and methods

### Study design and participants

In this prospective, controlled study, a total of 36 single segment painful acute osteoporotic vertebral compression fracture patients admitted into our department between September 2019 and February 2020 were enrolled. All participants were randomly divided into group A (PVP mainly assisted by three-dimensional-printed individual guide template) and group B (PVP mainly guided by C-arm fluoroscopy), the randomization ratio was 1:1 between group A and group B. The allocation was carried out as a block randomization, researchers, and participants were blinded from the allocation sequence, which was saved in sequentially numbered, opaque sealed envelopes. Only when the recruited patients were satisfied with all inclusion criteria and needed to perform the intervention, the envelopes containing details of the patients to be operated on were opened. An ethical clearance was obtained from the institutional ethical committee of the hospital. Patients’ informed written consent was obtained.

Two experienced spinal surgeons performed all operation procedures on these patients, and the operative materials, including puncture needles, bone cement, and percutaneous cement delivery system (Stryker Company, USA), are the same.

The present study was registered with the Chinese Clinical Trial Registry (ChiCTR) (http://www.chictr.org.cn), and its registration no. is ChiCTR1900024283.

### Inclusion criteria and exclusion criteria

The inclusion criteria are (1) presence of newly onset acute single segment OVCF; (2) no previous PVP treatment; (3) completed imaging data (including X-ray, MRI, CT); (4) VAS (visual analog scale) score higher than 6; and (5) patient request of operative treatment and refusal of conservative treatment.

The exclusion criteria are (1) patients with old OVCF; (2) patients with evidence of bone tumors and other bone metabolic diseases; (3) CT shows an incomplete or ruptured posterior wall of a fractured vertebral body; and (4) presence of surgical contraindication.

### Development of 3D printing guide templates

**Fig. 1:**
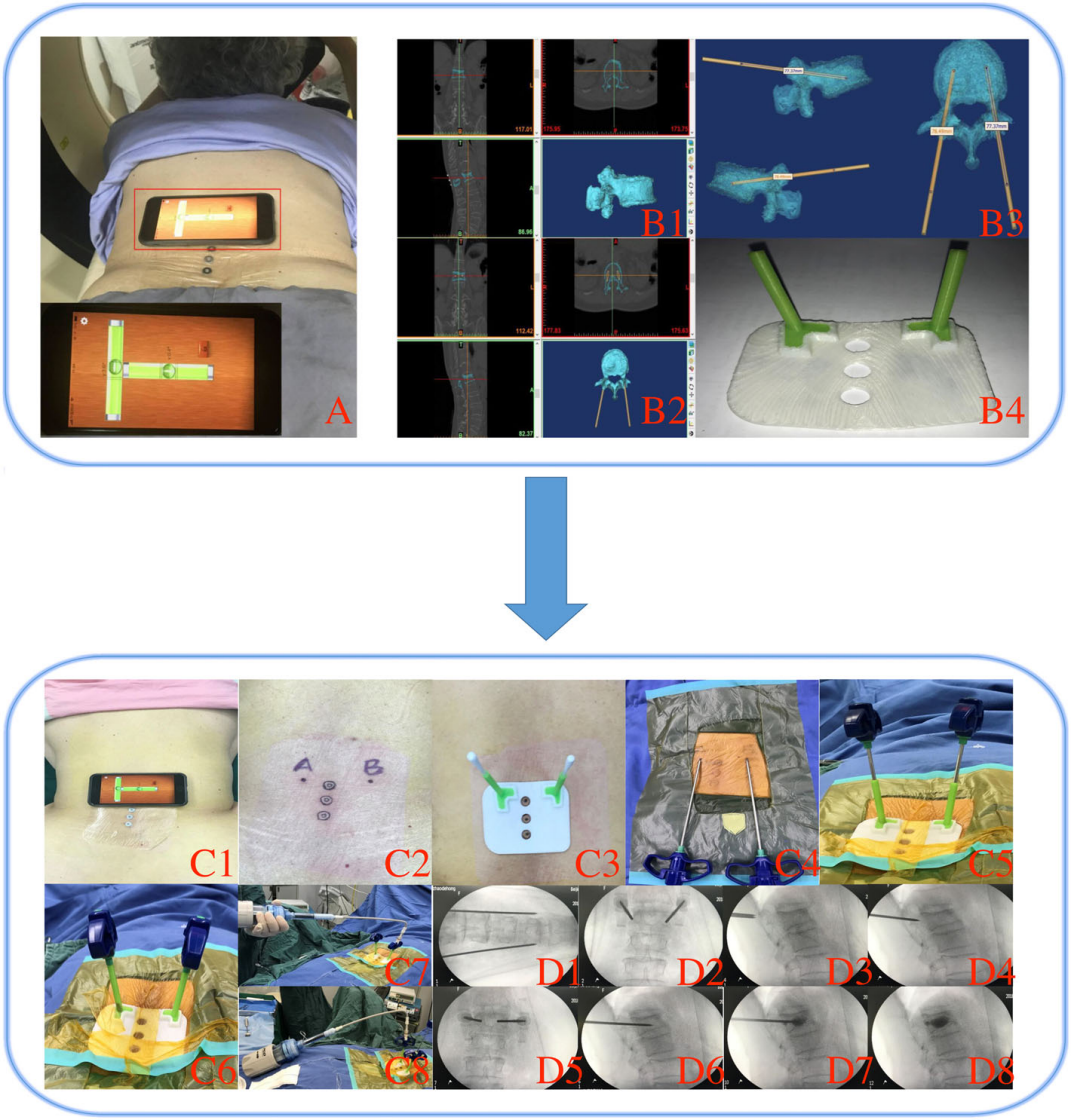
A case (no. 9 case in Table 1) in group A. A The patient did prone CT scanning with three markers located at the back skin near the fractured vertebra, the gradienter record the parameters of posture. B1 The reconstruction of the target vertebra in the coronal, transverse, sagittal plane in the MIMICS software, and the model of reconstructed vertebra. B2 The simulation of PVP in the MIMICS. B3 The parameters of guiding cylinders of the template. B4 The real template. C1 Using the gradienter to make sure the patient is in the same position when she did the CT. C2 Final puncture points. C3 Match one template with skin to determine the puncture points. C4 Using puncture needles to double check the puncture points. C5 Fixing the other sterilized template and insert the needles. C6 Tapping the needles to the end of the trajectories. C7–8 Injecting bone cement bilaterally via the needles. D1 One time fluoroscope for skin entry points. D2–6 With confirmation of fluoroscopy views, the needles were gradually tapped through the pedicle. D7 Injection of bone cement. D8 Fluoroscope for the check of the bone cement distribution

All of the patients in group A (PVP assisted by three-dimensional printing individual guide template) were conducted a prone computer tomography with three radiopaque markers (as the index markers for the template to attach to) placed in the midline of patient’s back skin at the compressed vertebral level. The patient’s CT scan body position was recorded by a gradienter on the patient’s back, while the patient was instructed to stay in the same position when doing the surgery (Fig. 1A). The CT images were saved in DICOM format, and the DICOM format files were exported into MIMICS (Materialise Interactive Medical Image Control System, Materialise Company) software for 3D reconstruction of the compressed vertebra (Fig. 1B1). After the target vertebra was reconstructed, we simulated a PVP procedure via a bilateral transpedicular approach in the software. First, the Medcad cylinder in the software was defined as the puncture needle model. Then, we simulated the entry point, the entry angle (head inclination angle and abduction angle orientation), and the puncture needle depth for a real PVP with the 3D views of the target vertebra. Next, we adjusted the puncture needles to its ideal position by using the move and rotate function (Fig. 1B2, B3).

After that, we saved all of the 3D template data and sent it in the MCS format to a three-dimensional printing company. They converted MCS data into STL format and applied 3-Matic software for computer-aided design for the template. They then used rapid prototyping technique to manufacture two same templates to sterilize for the operations (Fig. 1B4). The guide templates were made by polylactic acid, which can be sterilized by low-temperature steam disinfection.

The preoperative reconstruction of vertebra and simulation of PVP in MIMICS took about 30 min. We sent all the data to the 3D printing company 2 days before the operation to ensure the company could have sufficient time for the template printing and delivering template back to us.

### Procedures

The two groups of patients were all in the prone position during operations and underwent local anesthesia through the injection of a 5-ml mixture of 1% lidocaine and 1% ropavicaine. Each segment of the vertebra was injected 2 ml bone cement bilaterally and a total of 4 ml bone cement was injected.

### Group A operation procedures

Surgeons first made the patient lie prone on the operation table and maintain in the same position as for the CT examination in accordance with the gradienter record (no. 9 case in Table 1, Fig. 1C1). One template was matched with the target location according to the three radiopaque markers, and two swabs were inserted through the needle’s trajectories on the template (Fig. 1C3). The swabs were pressed to mark the insertion points on the skin, and drawn by a marker as point A and point B (Fig. 1C2). After the skin was disinfected, the surgical area was draped and put the tips of two puncture needles at the insertion points (Fig. 1C4). One shot of the anteroposterior view of C-arm fluoroscopy was used to confirm whether the puncture points determined by the template are the optimal insertion points (Fig. 1D1). After local anesthesia, another sterilized template was fixed on the patient's back by the sterilized film (Fig. 1C5). The two puncture needles were tapped into the target vertebra slightly via insertions through the guiding cylinders, and the C-arm fluoroscope was used to verify that the trajectories are suitable for insertion (Fig. 1D2, D3). After making sure the punctuation is within the pedicles, the needles were tapped to advance further until the end of the trajectories (Fig. 1C6, D3, D4). When the whole needles were completely inserted into the guiding cylinders, the C-arm fluoroscope was used to check if the needle tips have reached their ideal location (Fig. 1D5, D6). Bone cement was injected to the vertebra (Fig. 1C7, C8). Finally, the last two shots of anteroposterior and lateral views were performed to check the distribution of the bone cement within the vertebral (Fig. 1D8), and then the insertions were stitched.

**Table 1:** Patient clinical data

No.	Group	Age (years)	OVCF level	BMI(kg/m^2^)	Preoperative VAS	FTPP (times)	TRD (mGy)	TRT (times)	Operation time (min)	Cement leakage	Postoperative VAS
1	A	68	L2	24.30	8	1	4.77	16	18	No	3
2	A	59	L1	21.23	9	1	3.57	12	17	No	3
3	A	53	L1	27.22	8	2	4.47	15	19	No	2
4	A	62	T10	28.35	8	2	5.06	17	23	No	2
5	A	58	L1	22.72	8	1	4.47	15	18	No	3
6	A	65	T12	21.48	9	2	5.96	20	21	No	1
7	A	80	L3	24.65	9	1	5.36	18	21	No	2
8	A	68	L1	37.78	9	2	5.96	20	20	Yes	2
9	A	67	T11	28.88	8	1	3.57	12	17	No	2
10	A	55	L1	23.44	8	2	4.47	15	16	Yes	3
11	A	65	L1	19.56	8	2	6.55	22	21	No	2
12	A	64	T11	20.20	8	2	4.47	15	17	No	3
13	A	79	T12	23.61	9	1	4.77	16	19	No	3
14	A	71	T12	23.88	9	1	4.77	16	17	No	2
15	A	79	T6	19.98	9	4	4.77	16	20	No	3
16	A	66	L1	26.14	8	2	6.55	22	25	No	3
17	A	81	L3	26.12	8	3	5.36	18	20	Yes	2
18	A	61	L2	28.58	8	2	4.77	16	21	No	2
19	B	70	T12	23.44	9	5	8.64	29	26	Yes	1
20	B	80	T9	23.81	8	8	7.45	25	26	No	3
21	B	72	L4	28.69	9	4	7.45	25	29	No	3
22	B	53	T12	26.67	8	3	7.15	24	25	Yes	3
23	B	71	L4	32.05	8	6	11.02	37	32	No	3
24	B	70	T10	20.03	8	2	8.34	28	23	Yes	2
25	B	83	T12	21.48	9	6	6.26	21	22	No	2
26	B	61	T12	22.89	9	6	6.26	21	26	No	3
27	B	73	T12	27.36	9	4	6.26	21	26	No	2
28	B	58	L1	22.58	8	7	7.15	24	23	Yes	2
29	B	73	L1	24.61	8	5	8.34	28	27	Yes	3
30	B	85	T12	29.30	8	6	10.43	35	37	No	2
31	B	60	L1	25.39	9	8	8.64	29	31	No	3
32	B	75	T12	28.91	9	3	6.55	22	30	No	2
33	B	86	T11	17.35	9	8	11.02	37	35	Yes	3
34	B	76	T12	16.44	8	6	8.04	27	30	No	2
35	B	80	T12	28.06	8	3	6.85	23	25	Yes	2
36	B	67	T12	27.34	8	4	6.55	22	27	No	1

*Notes*: *Group A*, three-dimensional printing individual guide template-assisted PVP, *Group B* conventional PVP guided by C-arm fluoroscopy

*Abbreviations*: *FTPP* fluoroscopy times for puncture points, *TRD* total radiation dosages, *TFT* total fluoroscopy times, *TOT* total operation times, *VAS* visual analog scale

### Group B operation procedures

**Fig. 2:**
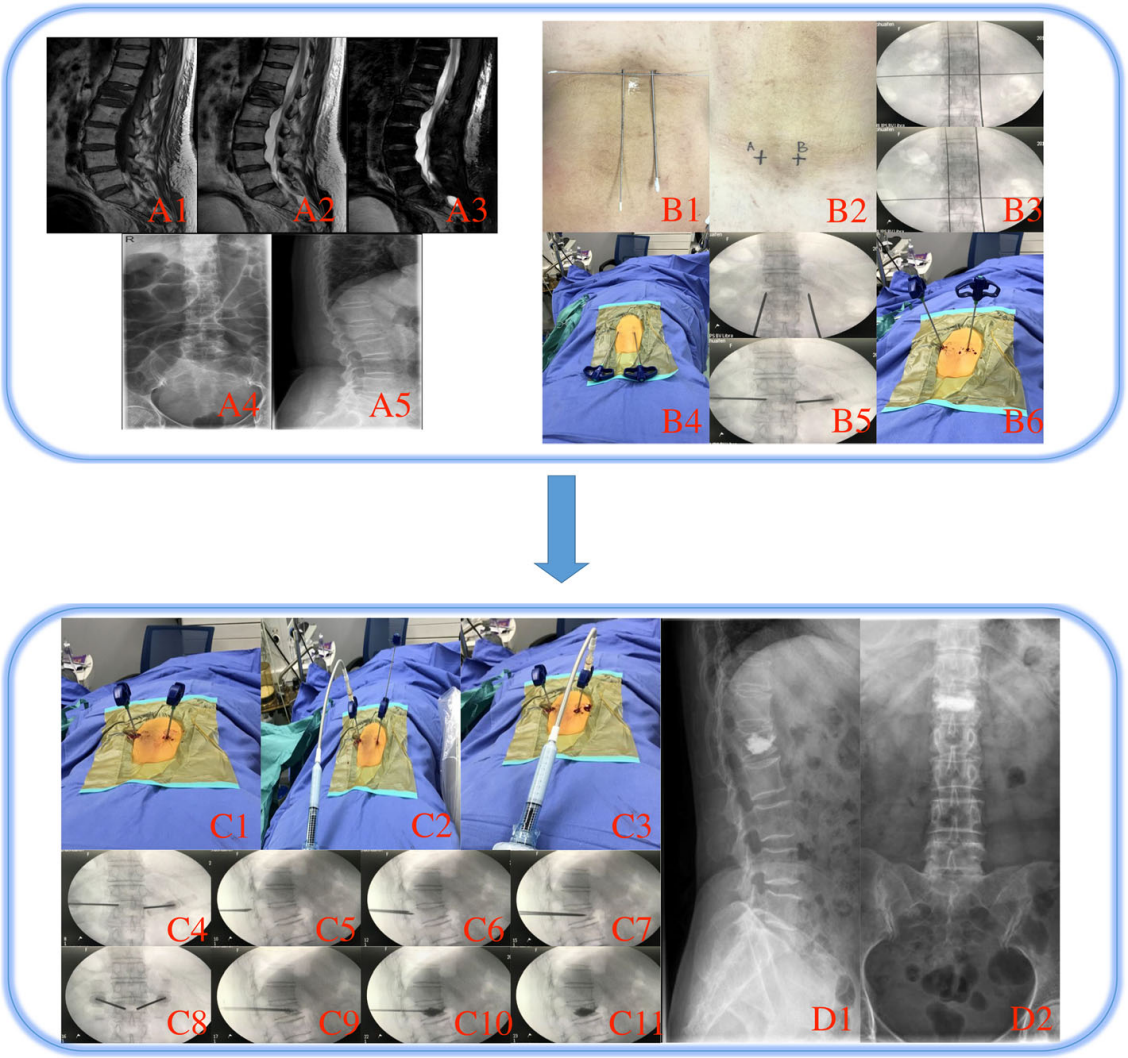
A case (no. 29 case in Table 1) in group B **A** The imaging of compressed vertebra. **A1** TIWI view. **A2** T2WI view. **A3** FS view. **A4** Posterioanterior view. **A5** Lateral view. **B1** Place Kirschner’s needles to determine the puncture points. **B2** Skin puncture points. **B3** Fluoroscope image of using Kirschner’s needles to determine the puncture points. **B4** Using puncture needles to double check the puncture points. **B5** Fluoroscope image of check the puncture points. **B6, C1** Tapping the needles for the punctuation. **C2–3** Injecting bone cement bilaterally via the needles. **C4-C8** With confirmation of fluoroscopy views, the needles were gradually tapped through the pedicle. **C9–C11** Injection of bone cement. **D1–2**) After the operation, lateral and anteroposterior view of fluoroscopy to check the bone cement distribution

Surgeons selected transpedicular bilateral approach for PVP. Kirschner wires were used to confirm the skin puncture points of bilateral pedicles of target vertebra under the anteroposterior fluoroscopy view (no. 29 case in Table 1, Fig. 2B1, B2, B3). To guarantee the punctuation with satisfied abduction angles of the puncture needles, the skin entry points were about 0.5–1.0 cm outside the superior-lateral points of bilateral pedicles. With the assistance of repeated fluoroscopy, surgeons adjusted the direction of the puncture needles to avoid the injury of the spinal cord, nerve roots, and found the optimal puncture trajectories (Fig. 2B4, B5, B6). Repeated fluoroscopy was used during the punctuation until the puncture needles reached the ideal location (Fig. 2C1–C6). Two shots of anteroposterior and lateral views fluoroscopy were performed to recheck (Fig. 2C7, C8). Bone cement was injected into the vertebra (Fig. 2C9, C10). Finally, the last two shots of anteroposterior and lateral views were performed to check the distribution of the bone cement within the vertebral body (Fig. 2C11) and stitched insertions.

### Outcome measure and statistical analysis

We collected all of the data of fluoroscopy times for puncture points (FTPP), total radiation dosages (TRD), total fluoroscopy times (TFT), pre-operative and post-operative visual analog scale (VAS) score, and total operation times (TOT) of both groups as the evaluation factors to evaluate two operation procedures. The post-operative radiographs of patients were used to check the leakage of bone cement. TFT and TOT are the main indicators to evaluate the accuracy and effectiveness of PVP assisted by three-dimensional printing individual guide template. FTPP means the number of X-ray fluoroscope that we used to determine the puncture points.

IBM SPSS 22.0 (SPSS Inc., USA) software was used for statistical analysis. Measurement data distribution was described in the mean ± SD (‾X±SD) form. A *P* value < 0.05 was considered statistically significant.

## Result

A total of 36 acute painful single segment osteoporotic vertebral compression fracture patients were recruited and randomly assigned to treatment groups. Patients’ data are shown in Table 1. One sample in group A (no. 6 in Table 1) was failed by the operation of PVP assisted by 3D printing guide template, then was finished by the conventional measure, and no complications happened on this patient. The surgery success rate in group A was 94.4%(17/18), the surgery success rate in group B was 100%(18/18).

## Clinical data

Group A consisted of 18 patients (2 males and 16 females) with a mean age of 67.1 ± 8.9 years (range 53–81) and mean BMI of 24.9 ± 4.4 kg/m^2^. Group B consisted of 18 patients (2 males and 16 females) with a mean age of 71.8 ± 9.4 years (range 53–86) and mean BMI of 24.8 ± 4.2 kg/m^2^. There were no significant differences between the two groups in terms of mean patient age, male/female ratio, and mean BMI (Table 2, *P* > 0.05).

**Table 2:** Comparative demographic and surgical data of both groups

	Group A	Group B	*P* value
Age (years)	67.1 ± 8.9	71.8 ± 9.4	*P* > 0.05
Male/female (*n*)	2/16	2/16	*P* > 0.05
BMI (kg/m^2^)	24.9 ± 4.4	24.8 ± 4.2	*P* > 0.05
Preoperative VAS	8.4 ± 0.5	8.4 ± 0.5	*P* > 0.05
FTPP (times)	1.8 ± 0.8	5.2 ± 1.9	*P* < 0.05
TRD (mGy)	4.9 ± 0.9	7.9 ± 1.6	*P* < 0.05
TFT (times)	16.7 ± 2.9	26.6 ± 5.3	*P* < 0.05
TOT (min)	19.4 ± 2.4	27.8 ± 4.0	*P* < 0.05
Postoperative VAS	2.4 ± 0.6	2.3 ± 0.7	*P* > 0.05
Complication incidence rate (%)	3/18, 16.7%	7/18, 38.9%	*P* > 0.05

The FTPP (1.8 ± 0.8 in group A vs 5.2 ± 1.9 in group B, *P* < 0.05), TRD (4.9 ± 0.9 vs 7.9 ± 1.6, *P* < 0.05), TFT (16.7 ± 2.9 vs 26.6 ± 5.3, *P* < 0.05), and operation time (19.4 ± 2.4 vs 27.8 ± 4.0, *P* < 0.05) were significantly different between group A and group B. There were no neurological complications or infections in both groups. No secondary surgical procedures were performed for any reason.

### Radiographic data

According to the postoperative radiography of patients, one physician who was blinded to the treatment group was asked to check the incidence of cement leakage. The results showed that the cement leakage occurred in group A (3/18, 16.7%) was less than that occurred in group B (7/18, 38.9%) (*P* > 0.05). All bone cement leakages are asymptomatic, which means the leakage did not induce spinal cord compression or any other neurological symptoms.

## Discussion

PVP was recommended by guideline as one of the most effective treatments for the back pain caused by OVCFs [22]. Nevertheless, some opinions point out that there is no significant difference in pain relief and quality of life enhancement between PVP and conservative treatment for acute OVCF patients [23]. However, Andrei [24] and Zhu [25] et al. hold the idea that PVP performs better than conservative treatment, which can rapidly relieve the pain caused by OVCFs. Before recruiting patients in the study, we informed all of them about the advantages and disadvantages of PVP and conservative treatment. All of the recruited patients requested for the PVP treatment refused conservative treatment.

With the rapid development of precision surgery, 3D techniques were increasingly applied in clinical practice to assist traditional operation procedures, including a 3D-printed model of bone fracture, reverse template, individualized implants, and so on [26, 27]. To treat OVCFs patients, a transpedicular approach guided by C-arm fluoroscope percutaneous vertebroplasty is the most commonly used technique [28]. To increase the success rate of traditional PVP procedure and to minimize the puncture-related complications, it is essential to determine the optimal puncture points, which requires repeated fluoroscopy. It is reported that inferior localization of puncture points of traditional PVP could lead to various kinds of complications [18]. Thus, in this study, our team designed and produced 3D printing guiding templates to assist the percutaneous vertebroplasty to improve the precision and safety of puncture technology of PVP, and got an ideal therapeutic effect.

The total operation time (TOT), fluoroscopy times for puncture points (FTPP), total radiation dosages (TRD), and total fluoroscopy times (TFT) were compared between group A and group B. The finding suggests that with the 3D-guided plate applied in the surgery, the average total operation time in group A was 19.4 ± 2.4 min, a statistically significant reduction from the total operation time in group B: 27.8 ± 4.0 min. In group A, the average fluoroscopy times during the operation were 16.7 ± 2.9 times, and the total radiation dosages were 4.9 ± 0.9 mGy, and the fluoroscopy times for puncture points were 1.8 ± 0.8 times. All of which were significantly lower than those in group B. These data proved that with the applied of 3D printing template, we turned traditional PVP into a more accurate and convenient surgical procedure, and it is indeed feasible to model a 3D individualized guide template to assist PVP procedure.

Comparing with the conventional C-arm fluoroscope assisted PVP, we concluded that there are some advantages in 3D-printed templates for assisting PVP: (1) a preoperative reconstruction of the target vertebra in the MIMICS software could let surgeons comprehensively grasp the morphology features of the fractured vertebra, and surgeons could make individualized punctuation trajectories in the software to simulate the punctuation of PVP. Modeling a patient-specific guide template and applying it during a real procedure could minimize the time of fluoroscopy during the operation, since the guiding cylinders on the template could predetermine the puncture needles’ orientations and depths. In contrast with the traditional way, we only need several fluoroscopes to check the accuracy of the guiding cylinders on the template, eliminating the needs of adjusting the puncture needles to determine the suitable entry points before surgery, furthermore, saving the time to adjust the direction and depth of the puncture needles during operation. Therefore, 3D-printed template could help shortening the PVP operation time, minimizing the fluoroscope times, and decreasing the radiation exposure for both doctors and patients during the procedure. (2) Through preoperative simulation of the transpedicular PVP approach in the software, we could establish optimal skin entry points, needle trajectories, insertion depths, and make the best puncture plan. Following the accurate puncture pathway, the incidence of cement leakage could be reduced. In our research, we could find that the incidence of cement leakage in group A (3/18, 16.7%) was significantly less than that in group B (7/18, 38.9%). Although there is no significant difference between the two groups, we suppose with the increase of recruited samples, it may present the statistical difference at the incidence of cement leakage. (3) Via preoperative simulation of the real operation, it turns the PVP from an experience-dependent operation method to a numerical, individual, precise operation method, which could minimize the risk of the procedure, as well as improving the accuracy and security of PVP. In this research, only single-segment vertebral compression fracture patients were included; we suppose there is a high possibility that a more valuable improvement would present on the complicated OVCF patients, like OVCF combine with scoliosis, kyphosis, vertebra rotation, or multiple segment OVCF. Even for experienced surgeons, performing those complicated OVCF operations still require multiple fluoroscopy scans and long operational times. (4) For young residents with fewer opportunity to perform the operation on patients, this technique may help shorten the learning curve of PVP and assist them to find the puncture points easier that needs further research.

**Fig. 3:**
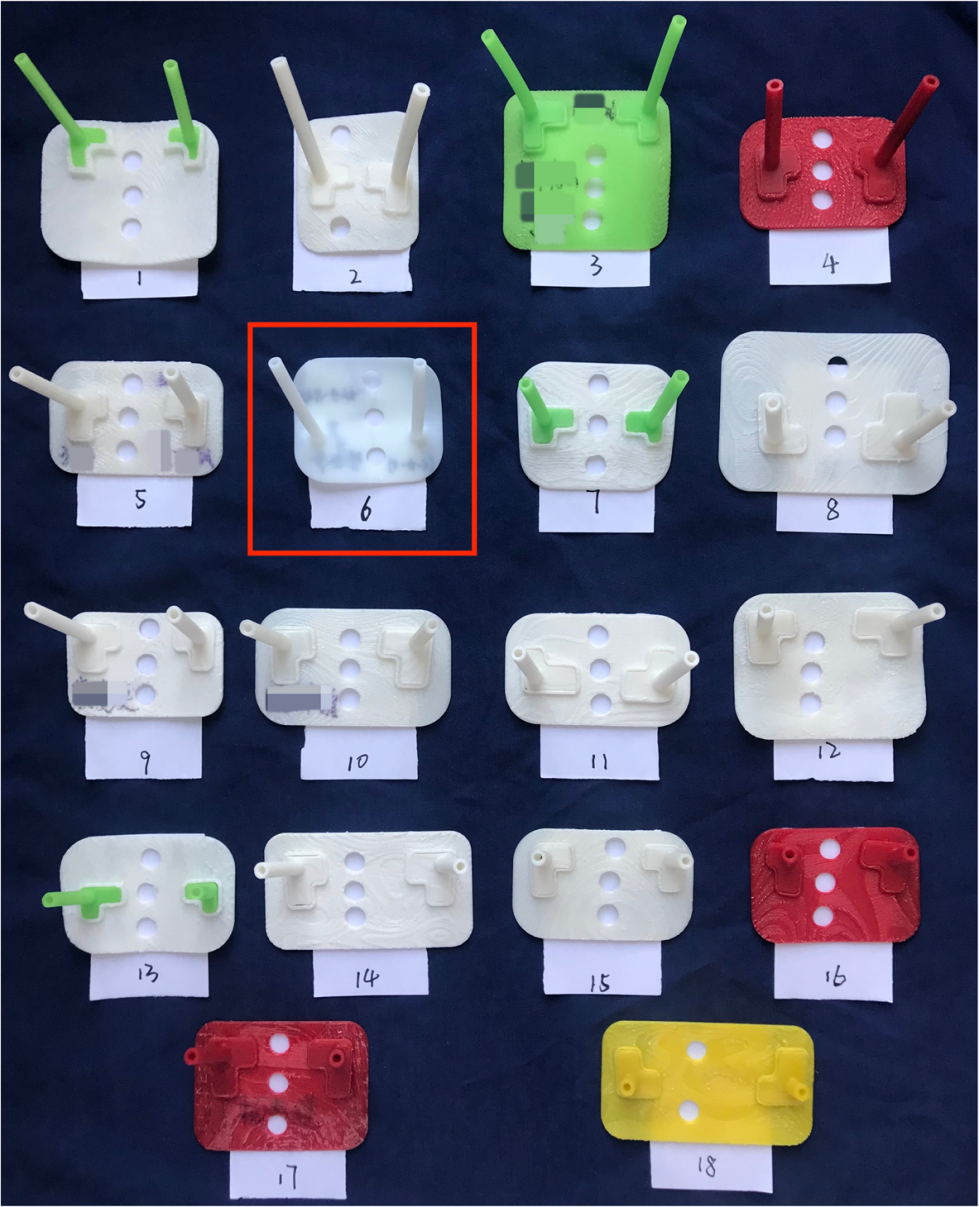
All of the templates in group A. No. 6 is the deformed template

In our study, one sample in group A failed by using 3D printing template to assist the operation. The guiding cylinders on the template along with the junction part of cylinders and base of the template got deformation after low-temperature steam disinfection (Fig. 3, no. 6); thus, the puncture needles could not perfectly pass through the cylinders to make punctuation. The main reason was in this suit of template, the guiding cylinders and the base of template were designed and integrated, instead of these parts could be separated and disinfected respectively. From this failed case, we concluded that template in this design type uses less material; it compromised the firmness of the template, so the template was so fragile for the disinfection. Therefore, in the rest of the cases, we designed the templates in the manner that cylinders and base were separated and could be disinfected respectively, so the usage of material could be sufficient enough to support the firmness of templates for the disinfection. Moreover, we are also exploring the other better materials to make templates more firm to replace the polylactic acid, which we are using now.

Besides, there are some other limitations of the application of a three-dimensional printing guide template in the PVP. (1) It takes time to master the use of the MIMICS software. During the template design, any single mistake made by surgeons unfamiliar with the software may lead to an unsuccessful surgery. Therefore, this method requires at least one surgeon in the team who is familiar with the software as well as the operation procedures. (2) Preoperative design of the template and template printing increased patients’ costs and the surgeon’s workload; each template will costs patient 1000 RMB.

## Conclusion

Collectively, 3D printing guide template-assisted percutaneous vertebroplasty is a more precise operation method compared to the traditional procedure. It could minimize the surgical time, radiation times, radiation exposure. This method also helps surgeons comprehensively visualize the fractured vertebra and develop an individualized surgical plan for the patient; therefore, we suppose it maybe more beneficial for complex cases. 

## Data Availability

All data generated or analyzed during this study are included in this published article.

## Abbreviations

PVP: Percutaneous vertebroplasty
OVCF: Osteoporotic vertebral compression fracture
FTPP: Fluoroscopy times for puncture points
TRD: Total radiation dosages
TFT: Total fluoroscopy times
TOT: Total operation times
VAS: Visual analog scale
